# Cross-Adaptation: Heat and Cold Adaptation to Improve Physiological and Cellular Responses to Hypoxia

**DOI:** 10.1007/s40279-017-0717-z

**Published:** 2017-04-07

**Authors:** Oliver R. Gibson, Lee Taylor, Peter W. Watt, Neil S. Maxwell

**Affiliations:** 1 Centre for Human Performance, Exercise and Rehabilitation (CHPER), Brunel University London, Uxbridge, UK; 2 Welkin Human Performance Laboratories, Centre for Sport and Exercise Science and Medicine (SESAME), University of Brighton, Denton Road, Eastbourne, UK; 3 Athlete Health and Performance Research Centre, ASPETAR, Qatar Orthopaedic and Sports Medicine Hospital, Doha, Qatar; 4 School of Sport, Exercise and Health Sciences, Loughborough University, Loughborough, UK

## Abstract

To prepare for extremes of heat, cold or low partial pressures of oxygen (O_2_), humans can undertake a period of acclimation or acclimatization to induce environment-specific adaptations, e.g. heat acclimation (HA), cold acclimation (CA), or altitude training. While these strategies are effective, they are not always feasible due to logistical impracticalities. Cross-adaptation is a term used to describe the phenomenon whereby alternative environmental interventions, e.g. HA or CA, may be a beneficial alternative to altitude interventions, providing physiological stress and inducing adaptations observable at altitude. HA can attenuate physiological strain at rest and during moderate-intensity exercise at altitude via adaptations allied to improved O_2_ delivery to metabolically active tissue, likely following increases in plasma volume and reductions in body temperature. CA appears to improve physiological responses to altitude by attenuating the autonomic response to altitude. While no cross-acclimation-derived exercise performance/capacity data have been measured following CA, post-HA improvements in performance underpinned by aerobic metabolism, and therefore dependent on O_2_ delivery at altitude, are likely. At a cellular level, heat shock protein responses to altitude are attenuated by prior HA, suggesting that an attenuation of the cellular stress response and therefore a reduced disruption to homeostasis at altitude has occurred. This process is known as cross-tolerance. The effects of CA on markers of cross-tolerance is an area requiring further investigation. Because much of the evidence relating to cross-adaptation to altitude has examined the benefits at moderate to high altitudes, future research examining responses at lower altitudes should be conducted, given that these environments are more frequently visited by athletes and workers. Mechanistic work to identify the specific physiological and cellular pathways responsible for cross-adaptation between heat and altitude, and between cold and altitude, is warranted, as is exploration of benefits across different populations and physical activity profiles.

## Key Points

**Table:** 

Adaptations to heat favourably reduce the physiological strain of subsequent hypoxia, largely via improvements to the cardiovascular system.
Adaptations to cold reduce the physiological strain of subsequent hypoxia by reducing sympathetic responses attenuating the autonomic responses.
At a cellular level, heat adaptations reduce the necessity to transcript further heat shock protein responses for cytoprotection, although the cellular adaptations to cold stress in subsequent hypoxia in humans remain largely unknown.

## Introduction

Athletic pursuits or occupational requirements can necessitate human exposure to a variety of environments. Human research has long considered the capacity for humans to respond and adapt to stressful environments, notably hot [1, 2] and cold conditions [3, 4], and low oxygen (O_2_) [high altitude] environments [5, 6]. These environments elicit stimuli-specific responses upon acute exposure, e.g. sweating in the heat [7], shivering in the cold [8] or elevating ventilation at altitude [9], with adaptation to each specific environment positively influencing these discrete responses. In addition to stimuli-specific physiological responses, environmental conditions also elicit shared physiological responses, e.g. acutely increased heart rate (HR) in cold [8], hot [7] and altitude environments [9]. Given the long-standing appreciation of these responses, it is surprising that work has only recently begun considering interactions between environment stressors in vivo in humans [10]. Research has begun to identify pathways by which generic- rather than environment-specific effector adaptations made in one environment can be physiologically beneficial within another. This work initially acknowledged that altitude-derived adaptations [11–15] can be beneficial during normothermic, sea-level exercise, an observation that now extends to heat-induced adaptations [16]. Recent reviews have proposed that heat-induced adaptations attained during heat acclimation/acclimatisation (HA) are physiologically beneficial in hypoxic/altitude environments [17–19], with experimental work now supportive of these hypotheses [20–23]. Conversely, less attention has been given to the role that cold adaptations attained during cold acclimation/acclimatisation (CA) [24] may have on the physiological responses to hypoxia/altitude, despite experimental evidence in humans also supporting this phenomenon [25].

It is the intention of this review to consider the body of evidence supporting thermal adaptations, i.e. those made in response to repeated heat or cold, on the subsequent physiological responses to hypoxia, a phenomenon that can be defined as cross-adaptation [26], as an alternative method for preparing individuals for altitude. At the present time, the paucity of data where environmental stressors are combined necessitates the exclusion of discussion allied to cross-adaptation between HA into cold, hypoxia stress, and CA into hot, hypoxic stress, although clearly HA retains relevance in hot, hypoxic environments, as does CA in cold, hypoxic environments.

### Cross-Adaptation Nomenclature

Within cross-adaptation (a generic term to describe the phenomenon) two distinct components to describe the induced changes have emerged—cross-acclimation/acclimatisation and cross-tolerance. Cross-acclimation (adaptation derived from a simulated environment [26]) or cross-acclimatisation (adaptation derived from a natural environment [26]) are adopted to define specific adaptations made at a physiological level due to commonality with typical descriptions of the acquired HA or CA phenotype [7, 8, 27]. Further to the use of cross-acclimation, cross-tolerance is the adopted nomenclature appropriate to describe cellular and molecular pathways relevant to adaptations between thermal and hypoxic environments due to common pathways with thermotolerance (also known as acquired cellular thermotolerance [28, 29]). While this nomenclature can be used in human and animal experimental work, it is the intention of this review to focus only on cross-adaptation experimental work conducted in humans.

## Heat to Hypoxia Cross-Adaptation

### Heat to Hypoxia Cross-Acclimation

#### Mechanisms by Which Heat to Hypoxia Cross-Acclimation May Occur

The heat-acclimated phenotype presents with improved effector heat loss mechanisms to overcome negative physiological responses to acute heat stress and consequently maintain internal endogenous temperature and central blood pressure [27] (see Sawka et al. [7] for a review of the negative physiological and performance responses to heat stress). The necessity to maintain blood pressure and perfusion to the exercising muscle, brain, and skin during heat stress results from an increased fraction of cardiac output (*Q*) distributed peripherally to the cutaneous circulation to facilitate heat loss to the environment [30–33]. At a physiological level, when under heat stress the maintenance of central and peripheral blood pressure is supported by a complex interaction of exercise- and temperature-induced hypervolaemia [34], specifically the plasma volume (PV) component of the blood [35], improved myocardial compliance [36], increased autonomic vasomotor control to regulate arterial and central venous pressure [37], sudomotor activation and efficiency [38], and reductions in onset thresholds for stimulating heat loss pathways [7]. The time course of each individual adaptation varies with a general trend for an increased magnitude of adaptation over time [39], with the reader directed to a meta-analysis describing the magnitude of heat adaptations in response to different HA regimes for further information [40]. Each of the described HA adaptations has the potential to favourably augment physiological responses to exercise in hypoxia, the most potent appearing to be those allied to cardiovascular, rather than thermoregulatory, physiology, given the likely compensable heat stress of most hypoxic/altitude environments [41]. The exception to this notion is when protective clothing/uniform is worn at altitude, since this will inhibit or mitigate evaporative heat loss, consequently posing a thermoregulatory challenge [42].

The proposal that HA might improve physiological responses and exercise performance in temperate conditions, a subtle form of cross-adaptation, has been discussed elsewhere [16], with debate questioning the magnitude of improvement in athletic performance in temperate conditions following HA [43–46]. The benefits for athletic performance remain equivocal [47–49]; however, various exercise-heat stress interventions, more logistically appealing than travelling to altitude, have been able to induce favourable physiological responses during exercise, e.g. HA [49–51], sauna exposure [52, 53], and hot-water immersion [54, 55]. These interventions induce adaptations, including PV expansion, reduced body temperature, improved blood distribution and enhanced exercise performance,, which may also prove beneficial in hypoxia [50, 52, 54]. Of these different strategies, HA remains the most well-understood, with the greatest volume of data supporting the attainment of desirable physiological adaptations [7, 27, 39, 40]. HA-derived PV expansion has been proposed as a fundamental mechanism to improve physiological responses and performance at altitude [19], with erythropoietic effects of hypervolaemia deemed unlikely [43], as recently demonstrated using the optimised carbon monoxide rebreathing technique during HA [20, 48].

Heat stress and hypoxia compromise O_2_ delivery to exercising muscle [56, 57], however different pathways are responsible. Under heat stress, reductions in $\dot{Q}$ distributed to exercising muscle is debilitative [32]. In hypoxia, despite equal or greater $\dot{Q}$ at a fixed exercise intensity, a reduced blood arterial oxygen content (CaO_2_)/partial pressure of oxygen (PO^−2^) entering the muscle concomitantly reduces exercise capacity due to the increased relative intensity of the task concurrent to a 6.3% decline in maximal oxygen uptake ($\dot{V}$O_2max_) with each 1000 m elevation in altitude [58]. It is noteworthy that the negative implications of altitude are further magnified with increasing aerobic training status due to increase muscular O_2_ utilisation [59] and incomplete pulmonary gas exchange with a higher $\dot{Q}$ [60]. In hypoxia the PO_2_ delivered to the muscle is reduced, becoming more pronounced with increasing ascents, and therefore limiting exercise performance [59]. Despite different limiting pathways in heat and hypoxia, the outcome is similar; reduced O_2_ is available for cellular respiration in the muscle, facilitating a decrease in the absolute maximal aerobic capacity and an increase in the relative intensity of a fixed task when performed in a normoxic, normothermic environment. Under conditions where O_2_ delivery is reduced, the body can maintain the required $\dot{V}$O_2_ at the muscle [32, 57] and brain [61, 62], via increased O_2_ utilisation. While increasing $\dot{Q}$ (O_2_ delivery) is a beneficial response to offset reductions in PO_2_ partially, this response is finite. The aforementioned dehydration experiments [32, 57, 61, 62] are an effective insight into the benefits of HA-induced PV expansion to increase $\dot{Q}$ and maintain O_2_ delivery in hypoxia.

#### Evidence of Heat to Hypoxia Cross-Acclimation

Comparisons between cross-acclimation experiments can be problematic, given the potential for the use of different HA protocols and durations, as the intervention, ineffective matching of control groups, timing of post-testing, and interindividual variation can each influence the observed adaptive response (see Taylor [27] for an overview of HA methods, and Tyler et al. [40] for a meta-analysis of the magnitude of adaptation relative to the timecourse of HA). Fortunately, at this early stage, cross-acclimation research has been largely consistent in the methods implemented, with similar timescales for the intervention employed. One difference exists in the use of fixed or isothermic HA protocols, although current evidence suggests these protocols generally possess similar adaptive capacities [51, 63].

Heled et al. [23] were the first to describe the effects of HA on exercise performance in hypoxia. In moderately trained humans, HA (12 × 120 min sessions of walking at 30% $\dot{V}$O_2max_) did not induce changes in $\dot{V}$O_2max_ (pre-HA [57.0 ± 3.7 mL kg^−1^ min^−1^] compared with post-HA [57.1 ± 2.9 mL kg^−1^ min^−1^]) at a moderate altitude (2400 m; fraction of inspired oxygen [FiO_2_] ≈0.156). However, peripheral oxygen saturation (SpO_2_%) did improve at 7 km h^−1^ (pre-HA 86.5 ± 2%; post-HA 88.0 ± 2%), and HR at the onset of blood lactate accumulation (4 mmol.L^−1^) during an incremental treadmill test (+1 km.h^−1^ every 3 min) was delayed (pre-HA [~160 b min^−1^] compared with post-HA [~170 b min^−1^]), suggesting that, although not specifically reported, typical HA phenotypic adaptations (e.g. PV expansion, improved myocardial compliance and reduced cardiovascular strain [40]) had likely occurred, and thus facilitated improvements in a moderate intensity domain (Table 1). Although providing seminal data at an altitude commonly used for training and competition [15], the exposure was relatively brief (<30 min), lacking an appropriate control group for comparisons with the experimental group, and the measured physiological responses to the intervention were minimal, therefore the findings have limited capacity for mechanistic interpretation, or application for populations who sojourn to altitudes for long durations.

**Table 1:** Heat to hypoxia cross-acclimation experimental data

Study, year	Heat acclimation protocol	Adaptations to heat acclimation	Hypoxic protocol	Improved responses to hypoxia
Heled et al. [23], 2012	12 days 120 min day^−1^ 40 °C 40% RH [Fixed] Walking @ 30% $\dot{V}$O_2peak_	Peak HR = ↓ 12 b min^−1^ Peak *T*_rec_ = ↓ 0.24 °C	OBLA test (FiO_2_ = 0.15; ~2500 m)	HR @ OBLA = ↓ 10 b min^−1^ SpO_2_ @ 7 km h^−1^ = ↑ 1.5%
Lee et al. [66], 2014	3 days 75 min day^−1^ (including 15 min preliminary rest) 40 °C 20% RH [Fixed] Cycling @ 50% $\dot{V}$O_2peak_	Heat tolerance = ↑ 3 min Sweat rate = ↑ 23% Plasma volume = ↑ 4.6%	15 min @ rest 60 min @ 50% $\dot{V}$O_2peak_ (FiO_2_ = 0.14; ~3300 m)	Peak HR = ↓ 9 b min^−1^ Mean HR = ↓ 9 b.min^−1^ Peak *T*_rec_ = ↓ 0.3 °C Mean *T*_rec_ = ↓ 0.2 °C Peak *T*_skin_ = ↑ 0.6 °C Mean *T*_skin_ = ↑ 0.9 °C
Gibson et al. [20], 2015	10 days 90 min day^−1^ 40 °C 40% RH [ISO] Cycling @ 65% $\dot{V}$O_2peak_	Resting HR = ↓ 18 b min^−1^ Resting *T*_rec_ = ↓ 0.49 °C Sweat rate = ↑ 48% Plasma volume = ↑ 14.7%	10 min rest 10 min cycling @ 40% $\dot{V}$O_2peak_ 10 min cycling @ 65% $\dot{V}$O_2peak_ (FiO_2_ = 0.12; ~4500 m)	HR @ 65% $\dot{V}$O_2peak_ = ↓ 12 b min^−1^ SpO_2_ @ 65% $\dot{V}$O_2peak_ = ↑ 3% $\dot{V}$O_2_/HR @ rest = ↑ 0.5 mL bt^−1^ $\dot{V}$O_2_/HR @ 65% $\dot{V}$O_2peak_ = ↑ 1.3 mL bt^−1^ RER @ rest = ↓ 0.06
White et al. [22], 2016	10 days 2 × 50 min day^−1^ (10 min rest interval) 40 °C 55% RH (1600 m altitude) [Fixed] Cycling @ 50% $\dot{V}$O_2peak_	Exercise HR = ↓ 21 b min^−1^ Exercise *T*_rec_ = ↓ 0.5 °C Plasma volume = ↑ 1.9% RPE = ↓ 3 TS = ↓ 1.0	16.1 km cycling TT (FiO_2_ ≈0.12; 4350 m)	TT time = ↓ 1.6% (*p* = 0.07)
Lee et al. [21], 2016	10 days 75 min day^−1^ (including 15 min rest) 40 °C 25% RH [Fixed] Cycling @ 50% $\dot{V}$O_2peak_	Mean HR = ↓ 14 b min^−1^ Resting *T*_rec_ = ↓ 0.26 °C Exercise *T*_rec_ = ↓ 0.54 °C Plasma volume = ↑ 3.5% Sweat rate = ↑ 88% TS = ↓ 0.9	40 min @ 50% $\dot{V}$O_2peak_, then 16.1 km cycling TT (FiO_2_ = 0.14; ~3300 m)	Mean HR = ↓ 9 b min^−1^ Mean SpO_2_ = ↑ 2% $\dot{V}$O_2_/HR = ↑ 0.7 mL bt^−1^ Mean *T*_rec_ = ↓ 0.15 °C TT time = ↓ 4.7%

*[Fixed]* denotes fixed-intensity protocol, *[ISO]* denotes isothermic/controlled hyperthermia protocol, *FiO*_*2*_ = fractional inspired oxygen content, *HR* heart rate, *OBLA* onset of blood lactate accumulation, *RH* relative humidity, *RER* respiratory exchange ratio, *RPE* rating of perceived exertion, *SpO*_*2*_ peripheral oxygen saturation, *T*_*rec*_ rectal temperature, *TS* thermal sensation, *T*_*skin*_ skin temperature, *TT* time trial, $\dot{V}$O_*2peak*_ peak oxygen uptake, ↑ indicates increase, ↓ indicates decrease

It has recently been observed that comparable HA regimes induce improved physiological responses [20, 21], and may [21], or may not [22], improve exercise performance in hypoxia. The difference between these comparable interventions is potentially the large (+14.7% [20]) to moderate (+3.5% [21]), or negligible (1.9% [22]), PV expansion (Table 1). It was first shown that 10 × 90 min sessions of isothermic HA attenuates physiological responses to hypoxia (FiO_2_ = 0.12 [20]). Additionally, post-HA, reductions in exercising (65% peak oxygen uptake [$\dot{V}$O_2peak_]) HR (−12 b min^−1^) in hypoxia in response to the same absolute workload were concurrent with improved SpO_2_ (+3%), and increased O_2_ pulse ($\dot{V}$O_2_/HR; +0.5 mL bt^−1^). Improvements in respiratory exchange ratio (RER) at rest (−0.06, *p* *=* 0.04) and a trend for improvements during exercise at 65% $\dot{V}$O_2peak_ (−0.05, *p* *=* 0.19) suggested a shift towards lipolysis had also occurred. These responses were improvements from equivalent pre-HA tests, and were in comparison with normothermic training controls. Mechanistically, a 14.7% expansion of PV and ~0.5 °C reduction in core temperature accompanied these responses. The authors concluded [20], in support of earlier reviews [17, 19], that while reduced core temperature was beneficial at facilitating a leftward shift in the oxyhaemoglobin dissociation curve, a more potent mechanism was likely to be the PV expansion due to the capacity for HA to maintain desirable rates of O_2_ perfusion at the exercising muscles at altitude.

Mechanistically increased PV facilitates maintenance of SpO_2_ following HA, with no apparent change in $\dot{V}$O_2_ [20]. This is likely due to reductions in HR and blood viscosity affording a greater erythrocyte alveolar transit time [60]. These post-HA responses are analogous to that observed in well-trained individuals possessing a typically larger *Q*, and reduced pulmonary gas exchange time leading to greater desaturation at higher exercise intensities [60]. A prolonged erythrocyte transit time facilitates a more complete resaturation within the pulmonary system [60, 64]. While also beneficial in normoxia [43, 44], the PV response is likely to be more pertinent in hypoxia due to further reduced SpO_2_. Support for a PV-mediated mechanism in cross-acclimation is derived from HA interventions that did not induce hypervolaemia, resulting in no significant improvements in aerobic exercise performance in hypoxia [22]. Some caution should be applied when interpreting experiments where PV is estimated absent of the measurement of red cell mass, i.e. those where ∆PV is determined based on calculations from measured haematocrit and haemoglobin concentrations only, rather than using a technique such as carbon monoxide rebreathing.

Over 10 × 100 min sessions of a fixed-intensity (~55% $\dot{V}$O_2max_) HA intervention [22], White et al. [22] observed that while not detrimental to performance in hypoxia, HA failed to provide more explicit evidence for cross-acclimation-derived performance improvements at altitude. In that experiment, only a 1.9% increase in PV and 1.6% improvement in performance (*p* *=* 0.07) was observed [22]. These data contrast work suggesting that HA reduces physiological strain in hypoxia [20, 21], and data by Lorenzo et al. [49], who demonstrated beneficial effects of HA in hot and cool conditions. The disparity in the results of the studies by Gibson et al. [20] and White et al. [22] does not entirely diminish confidence in the cross-acclimation response. Within the analysis of White et al. [22], it was reported that PV increased in five participants but decreased in three. Individual 16 km time trial (TT) data after HA from White et al. [22] showed that seven of eight subjects improved their TT performance, suggesting individual variation should be considered and that variation in PV expansion could have contributed to the trend towards a significant performance improvement in hypoxia. The duration required to complete the 16 km TT after HA decreased by 28 s, with magnitude-based inferences suggesting that the effect of HA was 96.5% ‘likely beneficial’ to performance at 4350 m, providing further confidence in the efficacy of the intervention [22]. Additionally, it is notable that participants were all residents at altitude of 1500–1600 m and likely partially acclimatised to the low O_2_. This may have also diminished the magnitude of response. Finally, while no difference in end-exercise HR, SpO_2_ and perceived exertion were observed following the HA intervention [22], this is not surprising as the power output was increased for these individuals. Had the participants performed a fixed workload task, it is likely that the physiological responses would be improved with HA, as observed by Gibson et al. [20].

**Fig. 1:**
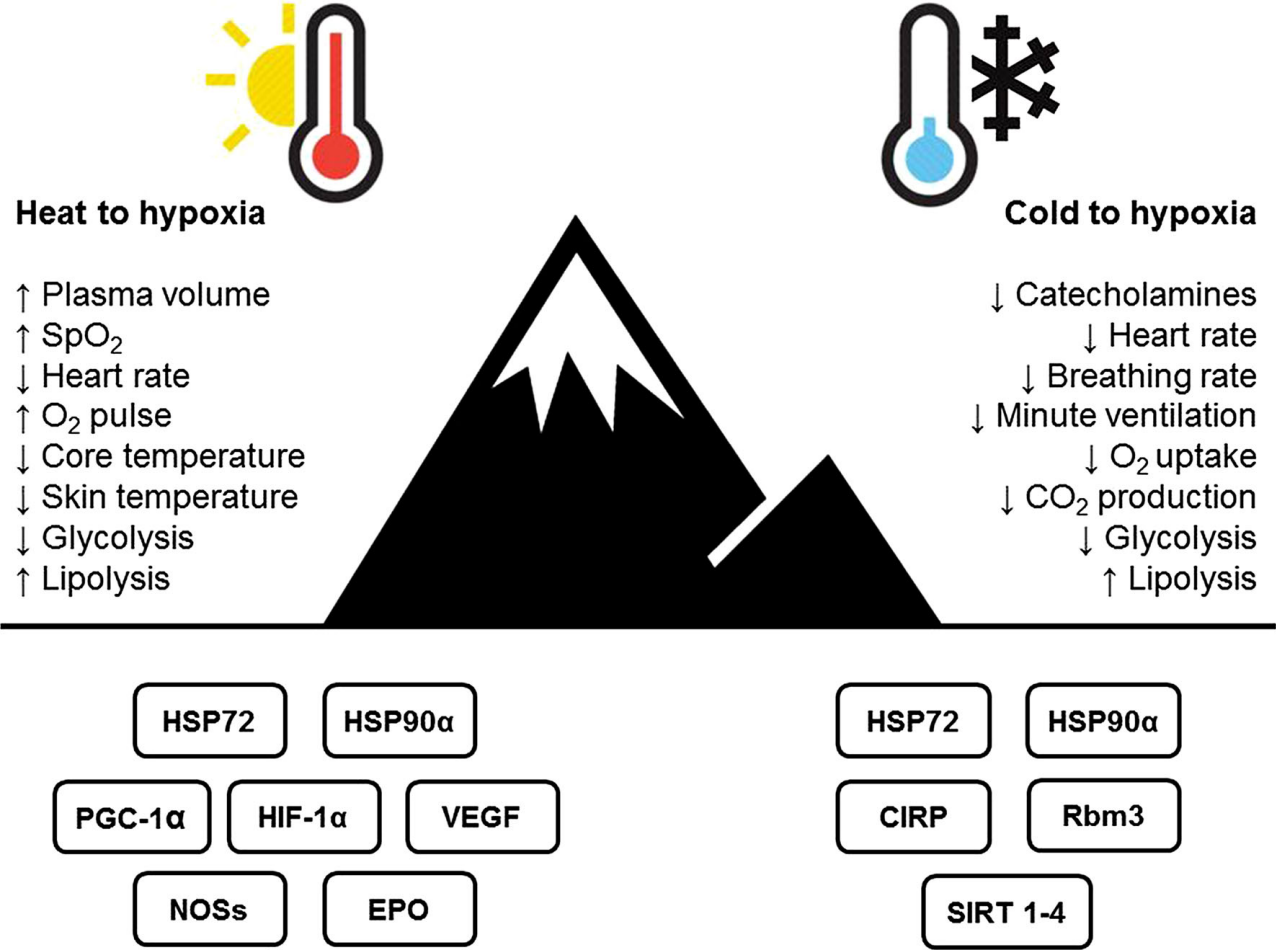
Identified mechanisms for cross-acclimation between heat and hypoxia (*left*), cold and hypoxia (*right*), and identified molecular targets relevant to cross-tolerance in hypoxia (*bottom*). *CIRP* cold-inducible RNA-binding protein, *CO*_*2*_ carbon dioxide, *EPO* erythropoietin, *HIF* hypoxia-inducible factor, *HSP* heat shock protein, *NOSs* nitric oxide synthases, *O*_*2*_ oxygen, *PGC* peroxisome proliferator-activated receptor gamma coactivator, *Rbm3* putative RNA-binding protein 3, *SIRT* sirtuin, *SpO*_*2*_ peripheral oxygen saturation, *VEGF* vascular endothelial growth factor, ↑ indicates increase, ↓ indicates decrease

 Some limitations exist that may generate individual variation in acclimation response, and mitigated the observation of undisputable evidence for cross-acclimation within the experiment [22]. These include the timing of the post-testing (8 days after HA), thus allowing PV expansion to decay [27], the pre-existing altitude habitation and training of participants, and implementation of a fixed, rather than isothermic, HA regime that maintains the potentiating stimuli for physiological adaptation to a greater extent [51, 63]. Regrettably, no control group was included by White et al. [22] to give confidence of a true HA versus training effect. Nonetheless, the hypoxic trials of White et al. [22] and Gibson et al. [20] were performed at comparable altitudes of 4000–4350 m and utilised comparable exposure durations (~30–40 min). Given the ‘trend for’ [22] and observed statistical significance [20], further work is warranted to elucidate the full physiological and performance improvements induced by HA in hypoxia. A summary of the identified mechanisms are presented in Fig. 1.

Lacking the inclusion of a matched normothermic, normoxic training group as found in the study by Gibson et al. [20], the study by White et al. [22] was also unable to determine differences between the adaptive capacity of HA and an equivalent altitude/hypoxic training protocol. This is essential to determine whether HA is a viable alternative to hypoxic/altitude training. This was addressed by Lee et al. [21], who utilised a 10-day, fixed-intensity (~50% $\dot{V}$O_2max_) HA intervention whereby participants performed 10 × 60 min sessions of exercise at a workload corresponding to 50% of their normothermic, normoxic $\dot{V}$O_2peak_. Participants performed training in one of three environments: the control group trained in normothermic normoxia (18 °C, 35% relative humidity [RH] FiO_2_ = 0.21); a second group trained in hot conditions, i.e. HA (40 °C, 25% RH FiO_2_ ≈0.21); and the third group trained in hypoxic conditions, i.e. hypoxic/altitude training (18 °C, 35% RH FiO_2_ = 0.14 [~3300 m]) [21]. No benefit in a post-training hypoxic stress test (40 min cycling at 50% of normoxic $\dot{V}$O_2peak_ in FiO_2_ = 0.14 [~3300 m]) was observed in control conditions; however, hypoxic training induced a reduction in exercising HR (−14 b min^−1^). HA elicited an equivalent reduction in exercising HR to that of hypoxic training (−14 b min^−1^). The HA-mediated reduction in HR was also observable at rest (−11 b min^−1^). Additionally, core temperature decreased (rest = −0.3 °C, exercising = −0.5 °C) to a similar extent of that of Gibson et al. [20] (see Table 1).

 These adaptations were congruous with those seen in other studies investigating the independent and combined responses to thermal (20 and 30 °C) and very low (610 m) and moderate altitude (2000 m FiO_2_ ≈0.17) over similar durations of exposure (10 × 60 min sessions) and at a similar relative intensity (60% $\dot{V}$O_2peak_) [65]. Performance during a 16.1 km TT in hypoxic conditions (FiO_2_ = 0.14, ~3300 m [21]) demonstrated that while no improvements were made following control training (+0.8%), hypoxic training (−6.9%) and HA (−4.8%), both improved performance without further increasing physiological strain. These data suggest HA is an effective alternative to hypoxic training.

Given that no change occurred in the normothermic, normoxic $\dot{V}$O_2peak_ test post-hypoxic training or HA, it is proposed that the HA and hypoxic training facilitated an improvement in the sustainable $\dot{V}$O_2_ or % $\dot{V}$O_2peak_ during the 16.1 km TT in hypoxia. The mechanism behind this positive adaptation following HA or hypoxic training appears allied to the improved SpO_2_ that follows these interventions. Different pathways may be responsible for this improvement, with HA (SpO_2_ +2%) utilising increased PV to facilitate greater available $\dot{Q}$ and, ultimately, central O_2_ distribution/delivery, while hypoxic training (SpO_2_ +1%) likely elicits adaptations within the muscle that augment more efficient O_2_ exchange and utilisation rather than haematological adaptations that are improbable utilising this altitude training modality (see Millet et al. [12] for a review of the area). Specific experimental designs to identify the precise mechanisms underpinning these performance benefits within hypoxia are discussed in Sect. 2.1.3.

Lee et al. [66, 67] provide an insight into the necessary HA dose to elicit cross-acclimation. In the first of two experiments, Lee et al. [66] implemented an acute intervention whereby participants exercised for 90 min at 50% $\dot{V}$O_2peak_ in four conditions: normothermic normoxia (FiO_2_ ≈0.21, 20 °C, 40% RH), hyperthermic normoxia (FiO_2_ ≈0.21, 40 °C, 20% RH), normothermic hypoxia (FiO_2_ ≈0.14, equivalent to ≈3000 m, 20 °C, 40% RH), or combined heat and hypoxia (FiO_2_ ≈0.14, 40 °C, 20% RH). These interventions, performed in a randomised order, preceded a 60 min hypoxic stress test (cycling at 50% $\dot{V}$O_2peak_) in normothermic hypoxia (FiO_2_ = 0.14). It was observed that no difference in physiological responses occurred in the subsequent hypoxic stress test (24 h post initial exposure), with the authors concluding that an acute intervention was insufficient to induce the requisite magnitude of adaptation. A follow-up experiment [67] extended understanding of responses beyond an acute exposure by extending the dose towards that of a short-term HA regime (3 × 60 min cycling at 50% $\dot{V}$O_2peak_ in either hot conditions, i.e. HA [40 °C and 20% RH] or normothermic conditions [20 °C and 40% RH]). A greater magnitude of physiological adaptation was induced following 3 days [67] versus 1 day [66] (see Table 1). During the hypoxic stress test (cycling at 50% $\dot{V}$O_2peak_), performed 24 h following the final HA or normothermic training session, it was observed that exercising HR decreased (~5 b min^−1^) and SpO_2_ was elevated (+1%) for a greater duration of the trial in the HA group versus controls. These data indicated that the HA group was more tolerant to the acute hypoxic stress than those who had performed normothermic training.

#### Future Directions in Heat to Hypoxia Cross-Acclimation

There is good evidence to support heat to hypoxic cross-acclimation [20, 21, 23, 67]. HA-induced adaptations are likely of benefit and are proposed as an alternative, or preferred, method for eliciting adaptation for those individuals who are initially less tolerant to hypoxia, or respond poorly to repeated exposures of hypoxia (precluding hypoxic adaptation), but respond well to heat stress. The application of cross-acclimation should therefore be investigated across a spectrum of both simulated and actual altitudes. This evidence could inform practice within sporting and occupational disciplines; however, at the current time, such applications are limited due to a paucity of data from these populations, across increasing severities of hypoxia and during differing work intensities. Consequently, further mechanistic evidence is required to inform applied practice relative to the actual experiences of travelling athletes [68] and at-risk workers [69] for whom cross-acclimation may provide an alternative to traditional altitude training regimes. Incremental terrestrial (or simulated) ascents of ~800 m (FiO_2_ = 0.187), ~1800 m (FiO_2_ = 0.167) and ~2800 m (FiO_2_ = 0.147), as utilised by others [58], are sufficiently different from one another to allow comparisons to be made experimentally, while also ecologically valid for application towards athletic and occupational cohorts. Accordingly, it is proposed that these altitudes should be tested in further cross-acclimation experiments to supplement the data described within this review at higher altitudes of ~3300 m (FiO_2_ ≈0.14 [21]), ~4200 m (FiO_2_ ≈0.12 [20]), and 4350 m (FiO_2_ ≈0.12 [22]). 

In addition to determining the efficacy of cross-acclimation across ascents, the benefits should also be considered across exercise intensity domains between individuals of higher and lower aerobic capacity. Current evidence highlights the ability for HA to reduce physiological strain at rest and within the moderate- to high-intensity domain (50–65% $\dot{V}$O_2max_ [20, 21]). There appears to be less evidence supporting reductions in physiological strain in the lower-intensity domains (<50% $\dot{V}$O_2max_ [20]), with a likely, but currently equivocal, understanding of the benefits at performance intensities [21, 22]. The magnitude of improvement in $\dot{V}$O_2max_ itself may [70] or may not [71] be dependent on O_2_ delivery, thus reducing the mechanistic appeal of cross-acclimation to increase $\dot{V}$O_2max_. Accordingly, HA may not improve $\dot{V}$O_2max_ at altitude as expansion of PV and associated increases in $\dot{Q}$ to increase O_2_ delivery at $\dot{V}$O_2max_ are less important than O_2_ utilisation in the mitochondria, a factor apparently unaffected by HA [27]. Calbet et al. [56] oppose PV-mediated benefits in hypoxia, since, following altitude acclimatization, acute PV expansion (+17%) had no additional effect on maximal Q, $\dot{V}$O_2max_ and exercise capacity at high altitude (5260 m). These data from Calbet et al. [56] suggest cross-acclimation may be more impactful at submaximal intensities as the data describing physiological responses to altitude following the PV expansion were improved during submaximal work (120 W, ~50–55% maximum power) [56]. However, some caution should be exercised in the interpretation of these results, given that participants in the experiment of Calbet et al. [56] were altitude acclimatized individuals, which parallels the work of White et al. [22], who noted that the potential for cross-acclimation to influence $\dot{V}$O_2max_ and exercise performance may be diminished in those habituated to altitude. The magnitude of cross-acclimation that can be attributed to reduced temperature and/or PV expansion could be investigated using an experimental design whereby acute reductions in core temperature and acute PV expansion, both in isolation and combination, replicate the observed adaptive responses to HA.

Once applications allied to continuous exercise utilising primarily aerobic metabolism are better understood, the efficacy of cross-acclimation benefits during repeated and/or intermittent sprinting may yield noteworthy applications in other disciplines of athletic performance, e.g. for team sports in which altitude elicits reductions in performance [72] of a similar magnitude to that of equivalent work in hot conditions [73]. There is also a necessity to determine timescales required to induce cross-acclimation between sexes, with females displaying a delayed temporal pattern during the attainment of HA [74]. The decay of cross-acclimation is unknown; at the present time, the only evidence available is limited to that of the decay of HA in hot conditions [75]. Benefits with varying intensities and movement patterns, and the identification of any sex differences, would give further context towards understanding who may benefit from cross-acclimation. 

Cross-acclimation research to date has primarily focused on cardiovascular mechanisms. Accordingly, there is a necessity to determine alterations in neuromuscular function, particularly when exercise performance is important. It has been observed that high altitude acclimatization reduces central, but not peripheral, fatigue, potentially via improved CaO_2_ and/or PO_2_ [76], and preliminary data indicate that HA may augment neuromuscular function [77]. Another area for consideration is the role of cross-adaptation in mitigating the decline in cognition that occurs during exposure to environmental extremes [78], such as heat [79–81] or hypoxia [82, 83]. The seminal experiment in the field of cross-adaptation [23] observed that, following HA, the number of false positives during a visual vigilance task in hypoxia reduced (−50%), while four choice reaction times improved (+5.3%) compared with pre-intervention baseline. Accordingly, in addition to exploring biological responses and mechanisms resulting in cross-adaptation, experimental work determining the potential to elicit favourable changes in simple and complex cognitive tasks in hypoxia following HA (and CA) is warranted [78]; however, appropriate control groups should be included.

A final area of interest regarding the practicality of heat–hypoxic cross-acclimation is the use of hot-water bathing [54, 55], or sauna exposure [52, 53], to induce adaptations. These interventions are generally available to all, and, accordingly, present few barriers to their implementation and may be especially desirable when an additional exercise load is undesirable, e.g. for athletes [68]. Also desirable for athletes and workers may be the rapid induction of cross-acclimation via twice daily HA [84]. It might also be noteworthy that, since beneficial cross-acclimation adaptations are becoming more readily observed from isolated HA interventions, a combination of HA in addition to traditional hypoxic exposure might reduce the duration required to acclimate to altitude [10], or provide a greater magnitude of adaptation [65]. A discussion of the mechanisms by which heat may facilitate hypoxic adaptation can be found in Sect. 2.2.1.

### Heat to Hypoxia Cross-Tolerance

#### Mechanisms by Which Heat to Hypoxia Cross-Tolerance May Occur

Cross-tolerance experimental work has identified heat shock proteins (HSP) as key components of the cross-stressor response. At the current time, experimental evidence dictates that of greatest interest for cross-tolerance are HSP72 [17, 85] and HSP90α [18], both of which demonstrate increases in basal levels following HA [21, 86] and in response to various hypoxic exposures with [21, 67] and without exercise [87–89].

First discovered in response to heat stress [90], the HSP70 family is present in two predominant isoforms: a constitutively expressed protein isoform HSC70 (HSP73), and a highly inducible ‘chaperone’ isoform HSP72 (HSPA1A/HSPA1B) [91]. Increases in intracellular HSP72 (iHSP72) are largely dictated by the transcription factor heat shock factor 1 (HSF1), which is translocated to the nucleus whereupon, binding to heat shock elements (HSEs), appropriate messenger RNA (mRNA) [*Hsp*] transcription occurs. Inhibition of HSF1 increases the susceptibility to acute in vivo thermal stress (i.e. heat stroke [92]), and similarly precludes procurement of optimal physiological adaptation to chronic thermal stress (i.e. heat-acclimated phenotype [93, 94]). Conversely, upon gene transcription via HSF1, large changes occur in the iHSP72 content of cells, notably following acute and chronic exercise [86, 95]. While numerous molecular signals are elicited in response to exercise [96, 97] and thermal stimuli [98], a common observation in both independent and combined heat and exercise models [86, 99, 100], and in independent and combined hypoxia and exercise models [87–89, 101], is the increase in HSPs [29]. Most relevant to HA-mediated cross-tolerance, it has been demonstrated that HSP72 increases in response to thermal stress [102], notably an increase deep body temperature [103], although others have also observed HSP72 protein translation as being responsive to training of a continuous or intermittent nature, whereby large changes in temperature are unlikely [104]. Exercise elicits numerous cellular and molecular stressors that, in isolation or in combination, behave as inductive stimuli for increases in HSP72 [105]. Stimuli that induce changes include, but are not limited to, whole body and local hyperthermia [106], oxidative stress/free radical formation [88, 107], substrate depletion [108], hypoxia/ischaemia [89], altered pH [109] and increased calcium concentration [110].

The initially identified role of HSP72 in humans was to provide cellular protection during heat stress. Emerging data have since identified that HSP72 is important in facilitating positive heat [93] and heat-independent adaptations [105] at a physiological and cellular level. HSPs facilitate maintenance of cellular and protein homeostasis, with regulatory roles in mitigating apoptosis, and facilitate recovery from and adaptation to stress [28, 111, 112]. The functional benefits of increased HSP have recently been discussed in detail elsewhere [99, 105, 113], alongside their specific roles in heat to altitude cross-tolerance [17–19]. Increases in HSP72 during subsequent heat stress improve tolerance to a hot environment by maintaining intestinal epithelial tight junction barriers [114, 115] by increasing resistance to gut-associated endotoxin translocation [116] and/or by reducing the systemic inflammatory response [85, 117]. These responses which ameliorate pathologies associated with heat illness [118] occur in conjunction with the induction of the HA phenotype [93]. Animal models in which transgenic overexpression of HSP72 is induced demonstrate improved markers of oxidative metabolism, e.g. mitochondrial number and function [119], although it is not known if this is a result of increased HSP72 per se, or generally increased activity levels (training) in response to the genetic modification. Should the former be observed in humans in hypoxia, this is an appealing mechanism whereby individuals due to visit altitude for training might benefit from a pre-altitude HA intervention to improve the physiological response to altitude (cross-acclimation), thus attenuating the immediate decline in training quality while also benefiting from improved adaptive capacity in response to altitude training at a cellular and molecular level (cross-tolerance).

It has been identified that both HSP72 and HSP90 increase the stability of hypoxia-inducible factor-1α (HIF-1α), and, in doing so, they may provide improved erythropoietic [89] and angiogenic responses [120, 121], which are of significance when training in hypoxia [122, 123]. Indeed, HSP72 has recently been identified as a specific regulator of angiogenesis [121, 124] and erythropoiesis [125, 126]. Further, as yet unexplored, benefits proposed by Ely et al. [17] are that increased HSP72 may improve endothelial barrier integrity in the cerebral and pulmonary microcirculation, thereby reducing the severity of altitude illness, with HSP90 acting as a cofactor in the production of nitric oxide synthase (NOS) to regulate vasodilatation, potentially reducing pulmonary hypertension [127]. Many of these proposed mechanisms for cross-tolerance require experimental support, particularly in human studies, the underlying concept being contingent on the potential for HA to increase HSPs, with the subsequent attenuation of a response indicative of a reduced cellular stress response. Additionally, HSPs provide only one known pathway for cross-tolerance to occur; however, many others may exist and thus the focus on HSPs in isolation might be considered a limitation of current experimental work.

#### Evidence of Heat to Hypoxia Cross-Tolerance

Although animal models suggest the maximum cross-tolerance benefits occur after ~4-week interventions [99, 100], the necessity for prolonged (>10 day) protocols identified as requisite for cross-acclimation in humans may not be true of cross-tolerance, at least in terms of providing some, if not total, cellular resistance to altitude. Lee et al. [66] observed an acute cellular preconditioning effect in response to a single prior exercise heat stress session during subsequent hypoxia. Post-exercise, monocyte HSP72 increased +107% in normothermic normoxia (FiO_2_ ≈0.21, 20 °C, 40% RH), +153% in hyperthermic normoxia (FiO_2_ ≈0.21, 40 °C, 20% RH), +126% in normothermic hypoxia (FiO_2_ ≈0.14, equivalent to ≈3000 m, 20 °C, 40% RH) and +161% in the combined stressors of hyperthermic hypoxia (FiO_2_ ≈0.14, 40 °C, 20% RH). The greater increase in the two hyperthermic conditions reflected the importance of larger, sustained changes in core temperature for HSP72 gene transcription [103]. The elevation was transient following normothermic normoxia, with a return towards resting values 24 h after the acute bout. However, the elevations remained 24 h after hyperthermic normoxia (+130%), normothermic hypoxia (+118%) and combined hyperthermic hypoxia (+131%). A reduced monocyte HSP72 induction during the hypoxic stress test following hyperthermic normoxia, normothermic hypoxia and hyperthermic hypoxia indicated that cellular tolerance to hypoxia had been conferred via the increased environmental-specific stress in the absence of reduced physiological strain. Exercise in normothermic normoxia was not sufficient to induce increases in HSP72.

A follow-up experiment extended the acute preconditioning response into a 3-day HA regime [67]. The initial hypoxic stress test elicited an equal increase in monocyte HSP72 between groups (controls +34%; HA +39%). As a result of the increased physiological stress of HA, basal HSP72 increased (+28%) following the 3-day intervention, but remained unchanged in the control group (+3%). In line with the hypoxic stress test that followed the acute intervention of Lee et al. [66], the control group demonstrated a monocyte HSP72 increase (+48%), suggesting cellular protection against the hypoxic stress was insufficient. In contrast, HA attenuated the previously increased monocyte HSP72 expression, demonstrating that a protective effect from HA-derived HSP72 was ameliorating the signal to increase HSP transcription and translation in hypoxia, thus inducing cross-tolerance [67].

Similar responses to that of the monocyte protein [66, 67] were observed, by Gibson et al. [20], in leukocyte *Hsp*72 mRNA in response to hypoxia (Table 2). In this experiment, where, during a 30 min hypoxic stress test (FiO_2_ = 0.12) consisting of a 10 min rest followed by 2 × 10 min bouts of exercise at 40% and 65% $\dot{V}$O_2max_, HA but not normothermic training mitigated the hypoxia-induced increase in *Hsp*72 mRNA (pre-HA +37%, post-HA −5%), but not leukocyte *Hsp*90α mRNA (pre-HA +23%, post-HA +14%) that had previously occurred during the hypoxic stress test performed prior to HA. This was a comparable direction of response to that of McClung et al. [86] in peripheral blood mononuclear cells undergoing a post-HA *ex vivo* thermal incubation for HSP72 (pre-HA +3.3-fold, post-HA +2.2-fold). However, McClung et al. [86] observed an attenuation in HSP90α (pre-HA +87%, post-HA no change from basal) not evidenced by Gibson et al. [20] during in vivo hypoxia. In addition to cross-acclimation and improved performance data, Lee et al. [21] observed that improved cellular tolerance to hypoxia was equally attainable utilising either HA or hypoxic training. Resting monocyte HSP72 increased in response to HA (+62%) and hypoxic training (+58%) to a similar extent, but was unchanged after thermoneutral, normoxic control training (+9%). Increased HSP72 following HA and hypoxic training led to an attenuated HSP72 response to hypoxic exercise in 40 min hypoxic stress tests (50% $\dot{V}$O_2peak;_ 18 °C, 35% RH FiO_2_ = 0.14). This response was comparable in direction, i.e. HA provided cross-tolerance to hypoxic stress (Table 2), but of a greater magnitude of response to that observed using a 10-day [21], rather than a 3-day, HA intervention in identical conditions [67]. These data emphasise that while acute [66] and short-term [67] cross-tolerance benefits are attainable, a dose response is apparent. The work of Gibson et al. [20], and Lee et al. [21, 66, 67] highlight that increased HSP72 attained during HA provides sufficient cellular protection to mitigate further increases in the protein in subsequent hypoxia.

**Table 2:** Heat to hypoxia cross-tolerance experimental data

Study, year	Heat acclimation protocol	Adaptations to heat acclimation	Hypoxic protocol	Improved responses to hypoxia
Lee et al. [66], 2014	3 days 75 min day^−1^ (including 15 min preliminary rest) 40 °C 20% RH [Fixed] Cycling @ 50% $\dot{V}$O_2peak_	↑ Basal monocyte HSP72 ↑ e*Hsp*72 pre/post session	15 min @ rest 60 min @ 50% $\dot{V}$O_2peak_ (FiO_2_ = 0.14; ~3300 m)	Monocyte HSP72 increase attenuated after heat acclimation ↑ eHSP72 maintained after heat acclimation
Gibson et al. [20], 2015	10 days 90 min day^−1^ 40 °C 40% RH [ISO] Cycling @ 65% $\dot{V}$O_2peak_	↑ *Hsp*72 mRNA pre/post session ↑ *Hsp*90α mRNA pre/post session	10 min rest 10 min cycling @ 40% $\dot{V}$O_2peak_ 10 min cycling @ 65% $\dot{V}$O_2peak_ (FiO_2_ = 0.12; ~4500 m)	Attenuated *Hsp*72 mRNA increase after heat acclimation ↑ *Hsp*90α mRNA maintained after heat acclimation
Lee et al. [21], 2016	10 days 75 min day^−1^ (including 15 min rest) 40 °C 25% RH [Fixed] Cycling @ 50% $\dot{V}$O_2peak_	↑ Basal monocyte HSP72 (comparable magnitude to equivalent training in hypoxia)	40 min @ 50% $\dot{V}$O_2peak_ then 16.1 km cycling TT (FiO_2_ = 0.14; ~3300 m)	Monocyte HSP72 increase attenuated after heat acclimation

*[Fixed]* denotes fixed-intensity protocol, *[ISO]* denotes isothermic/controlled hyperthermia protocol, *FiO*_*2*_ fractional inspired oxygen content, *HSP72/Hsp72* heat shock protein-72 (*eHSP* extracellular protein, *HSP* intracellular protein, *Hsp* gene). *Hsp*90α heat shock protein-90α, *mRNA* messenger RNA, *RH* relative humidity, *TT* time trial, $\dot{V}$ *O*_*2peak*_ peak oxygen uptake, ↑ indicates increase

#### Future Directions in Heat to Hypoxia Cross-Tolerance

In vivo, human experiments should further consider the kinetics of cross-tolerance in response to HA over varying timescales [51, 63], isolating the independent effects of exercise stimuli and environmental stimuli [103], and implementing post-intervention testing in hypoxic conditions using fixed absolute and fixed relative intensity prescriptions. Some presented experimental work does not include a post-intervention determination of $\dot{V}$O_2max_, e.g. Gibson et al. [20], and therefore it cannot be determined whether attenuated HSP responses are a result of a reduced relative intensity or whether the physiological and cellular adaptations induced by HA mitigated the hypoxic response. Future work should acknowledge this potential limitation.

The efficacy of cross-tolerance could be supported by a number of experimental designs utilising intracellular HSPs derived from a range of tissue sites allied to the specific function under investigation. From a mechanistic perspective, experimental work using hypoxic cell culture work similar to that where thermal incubation was performed [86] appears warranted to determine, in a well-controlled ex vivo environment, the time course/kinetics of cross-tolerance, either on a day-by-day [89] or pre/post HA basis [63]. Such ex vivo cell culture analysis would yield novel and important data regarding the capacity of cross-tolerance to induce cytoprotective cellular adaptation. The benefits of this research paradigm are mechanistic and well-controlled, yet they should be complemented by further in vivo analysis to offer a more ecologically valid understanding.

To clearly determine independent roles played by increased HSP in the development of cross-tolerance, manipulation of the intracellular protein content is likely required. Quercetin, a plant flavonol and an antioxidant, has been demonstrated as effective in attenuating increases in basal and inducible HSP72 during HA [93], while clinical trials and animal models support the use of the α-amino acid glutamine [115] or O-(3-piperidino-2-hydroxy-1-propyl) nicotinic amidoxime (BGP-15) as an HSP72 co-inducer [128]. These pharmacological interventions attenuate and elevate HSP72, respectively, providing an opportunity to isolate the independent effects of physiological and cellular adaptations in cross-tolerance/acclimation. These manipulations also facilitate the opportunity to explore specific benefits of cross-tolerance. This is pertinent as the precise benefits of the cross-tolerance-mediated reduction in HSP currently lack context, and experimental support for the effects of a reduced disruption to cellular homeostasis in subsequent hypoxia is unavailable.

It remains unknown what the minimum increase in HSPs relevant to elicit cytoprotection is. This is likely dependent on the nature of the stress presented; thus, for each of the described benefits, the necessary dose of HSP must be identified to determine which timescale of HA to apply. From a practical rather than mechanistic perspective, in spite of differences in the temporal patterning of HA which has implications for cross-acclimation [74], it appears that the transcription of HSP72 does not differ between sexes during HA [129]; however, translation into elevated basal protein, at least during normothermic training, may be inhibited in females [130]. Should the latter be further evidenced in an HA model, this presents limitations for cross-tolerance in females, with implications for female athletes or occupational workers due to experience an altitude sojourn. A final unknown consideration relates to the application of HA to individuals experiencing conditions akin to anoxia, such as free divers [131], who may additionally benefit from both cardiovascular and cellular adaptation [132–136].

## Cold to Hypoxia Cross-Adaptation

### Mechanisms by Which Cold to Hypoxia Cross-Adaptation May Occur

Data supporting the benefits of cold adaptations and subsequent improvements in the physiological responses to altitude are sparse. Indeed, to the authors’ knowledge, only one experiment testing this phenomenon in humans has been published to date [25]. A rationale for this paucity of data, beyond the acknowledgement that the area of cross-adaptation is in its infancy, is the limited number and magnitude of reported physiological adaptations in response to cold stress that are likely impactful at altitude. As discussed in Sect. 2.1.1, adaptations to heat stress have direct implications for exercise in hypoxia due to favourable maintenance of, or increases in, $\dot{Q}$. Increased $\dot{Q}$ is beneficial in hypoxia by offsetting reductions in PO_2_ and improving CaO_2_ at the desired organ. During cold stress, $\dot{Q}$ is not compromised, Accordingly, adaptations allied to this mechanism to defend blood pressure and blood/O_2_ distribution are not made following CA. Instead, following exposure to acute cold stress, a rapid sympathetic systemic response is initiated to defend body temperature, notably via vasoconstriction, and alterations in metabolism. This response is less prevalent during exercise in the cold [137]. It is known that exposure to cold [138] and altitude [11] increases circulating catecholamines, and this hormonal response has metabolic implications, notably those allied to glycolysis. Thus, the attenuation of this response with repeated cold [139] or prolonged hypoxia [140] may be a beneficial cold to hypoxic cross-acclimation adaptation.

 Cold adaptation can be divided into habituation, metabolic adaptation, and insulative adaptation [8]. Habituation is characterized by physiological changes induced following repeated cold exposures whereby the magnitude of response to subsequent stress is attenuated compared with the pre-exposure state. Within habituation, metabolic adaptation is characterised by a reduction in stress-induced increases in thermogenesis. Metabolic defence of body temperature is initially facilitated by shivering thermogenesis to increase metabolic heat production [141], with non-shivering thermogenesis occurring later via metabolism of brown adipose tissue [142]. The latter (non-shivering) contributes to a lesser degree than the former (shivering thermogenesis) [8]. Insulative adaptation is characterized by enhancing mechanisms that conserve body heat [143, 144], notably reducing thermal conductance at the skin to the environment via vasoconstriction. Metabolic adaptations to CA can be achieved in similar time frames to HA interventions (~5–14 days [8]); however, the utilisation of short-term CA protocols fails to elicit markers of insulative adaptation [145, 146]. Extensive protocols (90 min, 18 °C exposures, 5 days week^−1^, 8 weeks) are required to induce changes in this pathway for temperature defence [143, 144]. Accordingly, given the lack of cross-acclimation benefits (excluding cases of combined altitude and cold), and the prolonged duration required to elicit meaningful adaptation, the remainder of this section will focus on metabolic adaptations, driven by changes in autonomic activation to repeated cold stress, and the beneficial evidence for this cross-acclimation pathway in hypoxia.

Increased metabolic rates on exposure to acute cold stress attenuates with repeated exposures [137]. This has been demonstrated as occurring after single [147] and repeated exposures at rest [148], with improvements in exercise economy, i.e. reduced $\dot{V}$O_2_ and reduced RER attainable in the cold [149]. The optimal number of sessions for metabolic inhibition to passive cold stress is approximately six cold-water immersions (14 °C until core temperature = 35.5 °C) where an ~2 mL kg^−1^ min^−1^ reduction in $\dot{V}$O_2_ was observed, although significant reductions in metabolism may be obtained after two immersion days [148]. Regrettably, no data have been published evidencing changes in substrate utilisation, e.g. a reduced RER, with metabolic CA. A reduced RER, indicative of preferential fat oxidation, is an appealing cross-acclimation mechanism whereby, in addition to reductions in total energy expenditure at altitude following CA, reduced energy derived from carbohydrate would potentially spare this substrate for exercise performance.

### Evidence of Cold to Hypoxia Cross-Adaptation

Evidence for the transfer of autonomic and subsequent metabolic adaptations in response to repeated cold-water immersions to hypoxia in humans is limited to one experiment [25]. Lunt et al. [25] observed that 6 × 5 min immersions in cold (12 °C) versus thermoneutral (35 °C) water (twice daily over 3 days) reduced the sympathetic response to a 10 min bout of cycling at 100 W breathing hypoxic gas (FiO_2_ = 0.12). Reduced catecholamine (adrenaline and noradrenaline) responses followed the cold, but not the thermoneutral, intervention, indicating the sympathetic response to hypoxia had been attenuated. At a physiological level, this autonomic inhibition elicited reduced HR (−8 b min^−1^) and HR variability, as well as breathing rate (−5 br min^−1^), and, while tidal volume increased (+0.3 L), minute ventilation decreased (−3.7 L min^−1^). A reduction in $\dot{V}$O_2_ (−0.1 L min^−1^), compared with the thermoneutral group, was observed, and reductions in hyperventilation reduced carbon dioxide production ($\dot{V}$CO_2_; −0.5 L min^−1^) and resulted in a reduction in RER (−0.1) (see Fig. 1). These metabolic responses are beneficial for those travelling to altitude, given that classical responses to hypoxia include increased ventilation, energy expenditure, and preferential use of glycogen [11]. The populations who would derive the greatest benefits would be those performing prolonged aerobic work at altitude [150], i.e. in a similar exercise-intensity domain, albeit for longer durations, to that experimentally investigated by Lunt et al. [25]. In addition to physiological adaptations, subjective responses to hypoxia also improved following cold-water immersion (−2 symptoms; −5 cumulative symptom severity). This observation, supported by data evidencing cognitive task improvements following a repeated cold-water immersion of a similar duration to that of HA [151], may be important when the performance of cognitive tasks at altitude is necessary.

### Future Directions in Cold to Hypoxia Cross-Adaptation and Cross-Tolerance

Given evidence that cold to hypoxic cross-adaptive pathways are primarily effective at reducing the sympathetic response to the environmental stimuli, the rapid attainment of this phenotype warrants future investigation. A multi-exposure, within-day series of cold treatments may accelerate the desensitisation of the autonomic and metabolic responses to the stress, a process that is not as time-dependent as adaptive processes congruent with HA or insulative CA, and which likely confers minimal benefits in hypoxia. The work of Lunt et al. [25] provides experimental evidence for the efficacy of cross-acclimation in a low- to moderate-intensity domain. It remains unknown whether this extends to increasing exercise intensities, including those that characterise athletic performance, or during chronic hypoxia. As with HA, the decay of CA into cross-acclimation is unknown; however, data suggest the effects of CA (two 3-min head-out immersions in water of 10 °C, 4 days apart) may last 14 months, which has clear appeal to those requiring intervention [152]. Animal models provide supporting data for the autonomic and metabolic pathways for future human study in the area (see Chauhan et al. [153] for a review).

 Cold cross-tolerance in humans is a novel area in which, to the authors’ knowledge, no human experimental work has been published. Accordingly, the role of ‘cold shock proteins’ and HSPs in response to CA remains a novel area for future investigation in humans despite similar cross-tolerance response genes, e.g. HSPs and sirtuins 1–4, in mammals [98] and fish [154]. Of interest are cold-induced RNA binding protein (CIRP) and RNA-binding motif protein 3 (Rbm3), which respond to both cold and hypoxia [155]. This cellular response may serve a novel cross-tolerance role in hypoxia, given that data identifying increases in the aforementioned cold shock proteins are consistent with associated reduced cellular damage during hypoxic or cold stress [156, 157].

 Short cold-air exposures also increase HSP72 expression compared with an equivalent thermoneutral control [158]. Accordingly, it is likely HSP72 expression is increased during/following repeated cold-water immersions. Mechanistically, this may not result from the cold per se, but be mediated by the indirect action of catecholamines on HSE and HSF1 [159], a mechanism to be further investigated.

Cold exposure, most effectively implemented via cold-water immersion, provides a simple and expedient method to improve physiological responses to hypoxia in populations such as athletes and occupational groups. The use of cold-water immersion e.g. ‘ice bathing’ is widespread in the athletic performance-recovery domain [160], indicating that the intervention is feasible for many sporting, and indeed occupational, applications. Caution should be exercised in those with contraindications to cold exposure and in those wishing to increase training status. While the risks to health following cold exposure are well-established for some [161], it has been shown that cold-water immersion post continuous or intermittent exercise is beneficial, acutely [162, 163] and with repeated use [164], with benefits linked to reduced body temperatures, consequently ameliorated central nervous system-mediated fatigue, and by reducing cardiovascular strain with enhanced parasympathetic activation [165]. Accordingly, a future research direction should investigate normal, scheduled training in the morning with subsequent cold exposures in the late afternoon or evening once recovery from the exercise has largely been facilitated. This method has potential additional benefits of improving sleep quality [166] and providing a reduction in thermal strain during ensuing exercise bouts [167], with some data showing that cold-water immersion following high-intensity interval training in the heat is able to further increase gene transcripts allied to mitochondrial biogenesis and the HSP response [168]. There is potential for cold-water immersions to ‘blunt’ training adaptation, however this appears more relevant to resistance training paradigms [169, 170], when implemented immediately post-exercise [171], a modality that is highly unlikely to be used for cross-adaptation. It is useful that the heat-hypoxia and cold-hypoxia cross-acclimation pathways are different. To induce the maximal cross-adaptive effect, it is further proposed that morning HA could be supplemented with evening cold-water immersion to elicit favourable cardiovascular, thermoregulatory, metabolic and cellular cross-adaptive responses; however, this proposition requires experimental elucidation to confirm our hypothesis.

## Conclusions

This review has highlighted the physiological and cellular (HSP) adaptations that can be induced using HA or CA to attenuate the disruptions to homeostasis upon exposure to hypoxia-inducing conditions. Although in its infancy, experimental data in the field should encourage further investigation of the benefits of cardiovascular and thermoregulatory adaptations via HA, and metabolic adaptations via CA, as cross-acclimation pathways effective across a spectrum of exercise intensities at various altitudes. Cross-tolerance via increased HSPs requires significant further investigation implementing well-controlled in vivo and ex vivo experimentation whereby the precise effects of accumulated basal HSPs on the specific negative effects of hypoxic stress and altitude illness are determined. Finally, once the precise mechanisms for cross-adaptation are identified, refinements to the methods implemented for each intervention should be made to optimise their use.
